# Role of 5-HT_7_ receptors in the immune system in health and disease

**DOI:** 10.1186/s10020-019-0126-x

**Published:** 2019-12-31

**Authors:** Alejandro Quintero-Villegas, Sergio Iván Valdés-Ferrer

**Affiliations:** 1 Escuela de Medicina, Universidad Panamericana, Mexico City, Mexico; 2 Departments of Neurology, Instituto Nacional de Ciencias Médicas y Nutrición Salvador Zubirán, Mexico City, Mexico; 3 Departments of Infectious Diseases, Instituto Nacional de Ciencias Médicas y Nutrición Salvador Zubirán, Mexico City, Mexico; 4 Center for Biomedical Science, Feinstein Institute for Medical Research, Manhasset, NY USA; 5 Department of Medicine, Intituto Nacional de Ciencias Medicas y Nutricion Salvador Zubiran, Mexico City, Mexico

**Keywords:** 5-HT_7_ receptors, Signaling pathway, 5-HT_7_ effect, 5-HT_7_ distribution, Inflammation, Dendritic cell, Microglia, macrophages, Lymphocytes

## Abstract

In mammalians, serotonin (5-HT) has critical roles in the central nervous system (CNS), including mood stability, pain tolerance, or sleep patterns. However, the vast majority of serotonin is produced by intestinal enterochromaffin cells of the gastrointestinal tract and circulating blood platelets, also acting outside of the CNS. Serotonin effects are mediated through its interaction with 5-HT receptors (5-HTRs), a superfamily with a repertoire of at least fourteen well-characterized members. 5-HT_7_ receptors are the last 5-HTR member to be identified, with well-defined functions in the nervous, gastrointestinal, and vascular systems. The effects of serotonin on the immune response are less well understood. Mast cells are known to produce serotonin, while T cells, dendritic cells, monocytes, macrophages and microglia express 5-HT_7_ receptor. Here, we review the known roles of 5-HT_7_ receptors in the immune system, as well as their potential therapeutic implication in inflammatory and immune-mediated disorders.

## Introduction

Serotonin (5-hydroxytryptamine [5-HT]), a monoamine neurotransmitter discovered over seven decades ago as a vasoconstricting agent (Rapport et al. 1948), has critical -and well defined- roles in the central nervous system (CNS), including regulation of mood stability, pain tolerance, or sleep patterns to name a few. Serotonin receptors are expressed throughout the immune system (Herr et al. 2017; Ahern 2011). 5-HT_7_ receptor is a member of the family of serotonin receptors, originally cloned in 1993 (Bard et al. 1993; Lovenberg et al. 1993; Ruat et al. 1993) a little more than a decade after the first receptor, 5-HT_1_ receptor, was (Peroutka and Snyder 1981). Like other serotonin receptors, 5-HT_7_ receptors are members of the G protein-coupled receptor superfamily. Their activation leads to the initiation of two well-characterized signaling pathways: the *canonical* signaling occurs through Gαs, while a *non-canonical* pathway signals through Gα12 (Guseva et al. 2014b). Different 5-HT_7_ receptor isoforms have been described (5-HT_7a_, 5-HT_7b_, 5-HT_7d_, all expressed in humans and rats, as well as 5-HT_7c_, expressed only in rats) differing only on the carboxy terminus length, nonetheless, no relevant functional differences have been observed between them (Liu et al. 2001).

### Distribution of 5-HT_7_ receptors

5-HT_7_ receptors are expressed mainly in two compartments: the CNS (Hedlund and Sutcliffe 2004) and the gastrointestinal (GI) tract (Yaakob et al. 2015), although they are also expressed in other tissues including immune cells (see below). In the CNS, the receptor is broadly expressed in the spinal cord, suprachiasmatic nucleus of the hypothalamus, antedorsal thalamus, globus pallidus, prefrontal cortex, trigeminal nucleus caudalis, raphe nuclei area, amygdala and hippocampus, particularly in pyramidal cells of *cornu ammonis* (CA)1 and CA3, where they are expressed in both, neurons and glial cells, including microglia, the CNS-specific phagocytic cell (Chapin and Andrade 2001; Dogrul and Seyrek 2006; Gill et al. 2002; Horisawa et al. 2013; Thomas and Hagan 2004; Tokarski et al. 2003; Russo et al. 2005; Hedlund and Sutcliffe 2004; Lovenberg et al. 1993).

5-HT_7_ receptors are found on smooth muscle cells in several arteries, including the aorta, cerebral, coronary, and pulmonary arteries, where their primary known role is to induce vasodilation (Jasper et al. 1997; Nilsson et al. 1999; Jähnichen et al. 2005; Chang Chien et al. 2015; Terrón and Falcón-Neri 1999). In the GI tract, 5-HT_7_ receptors are expressed not only in gut-associated neurons, but also in enterocyte-like and immune cells in lymphatic tissues scattered all along the gut (Iceta et al. 2009; Kim et al. 2013; Guseva et al. 2014b), including monocytes, lymphocytes and dendritic cells (DCs) (León-Ponte et al. 2007; Wu et al. 2019), where it may play a crucial role in inflammation signaling (Urbina et al. 2014; Soga et al. 2007; Holst et al. 2015). Also, 5-HT_7_ receptors are found in neutrophils, but the net effect of 5-HT_7_ receptor signaling and modulation on neutrophil function is yet to be defined (Rapalli et al. 2016). 5-HT_7_ receptors have also been identified in hepatic stellate cells and hepatocytes (Ruddell et al. 2006; Svejda et al. 2013).

Of direct relevance to the present review, 5-HT_7_ receptors are also present in immune tissues, including the spleen and thymus; peripheral blood (Stefulj et al. 2000); DCs and other bone marrow-derived mononuclear cells (Shen et al. 1993; Vanhoenacker et al. 2000; Idzko et al. 2004).

## Methods

We performed a comprehensive search of English language literature to identify all original research, and review articles regarding 5-HT_7_ receptors, signaling pathways and the effects on the immune system; PubMed database since 1993 was used. We used the following Medical Subject Headings (MeSH) and main keywords for searches: 5-HT_7_, LP-211, LP-44, LP-21, AS-19, SB 269970, 5-HT_7_ physiology, 5-HT_7_ receptor mechanism of action, 5-HT_7_ receptor signaling pathway, 5-HT_7_ receptor effect, 5-HT_7_ receptor distribution, inflammation, dendritic cell, microglia, macrophages, and lymphocytes. We also reviewed the reference lists of the articles identified during the search. The authors independently reviewed the selected articles.

## Signaling pathways

**Fig. 1:**
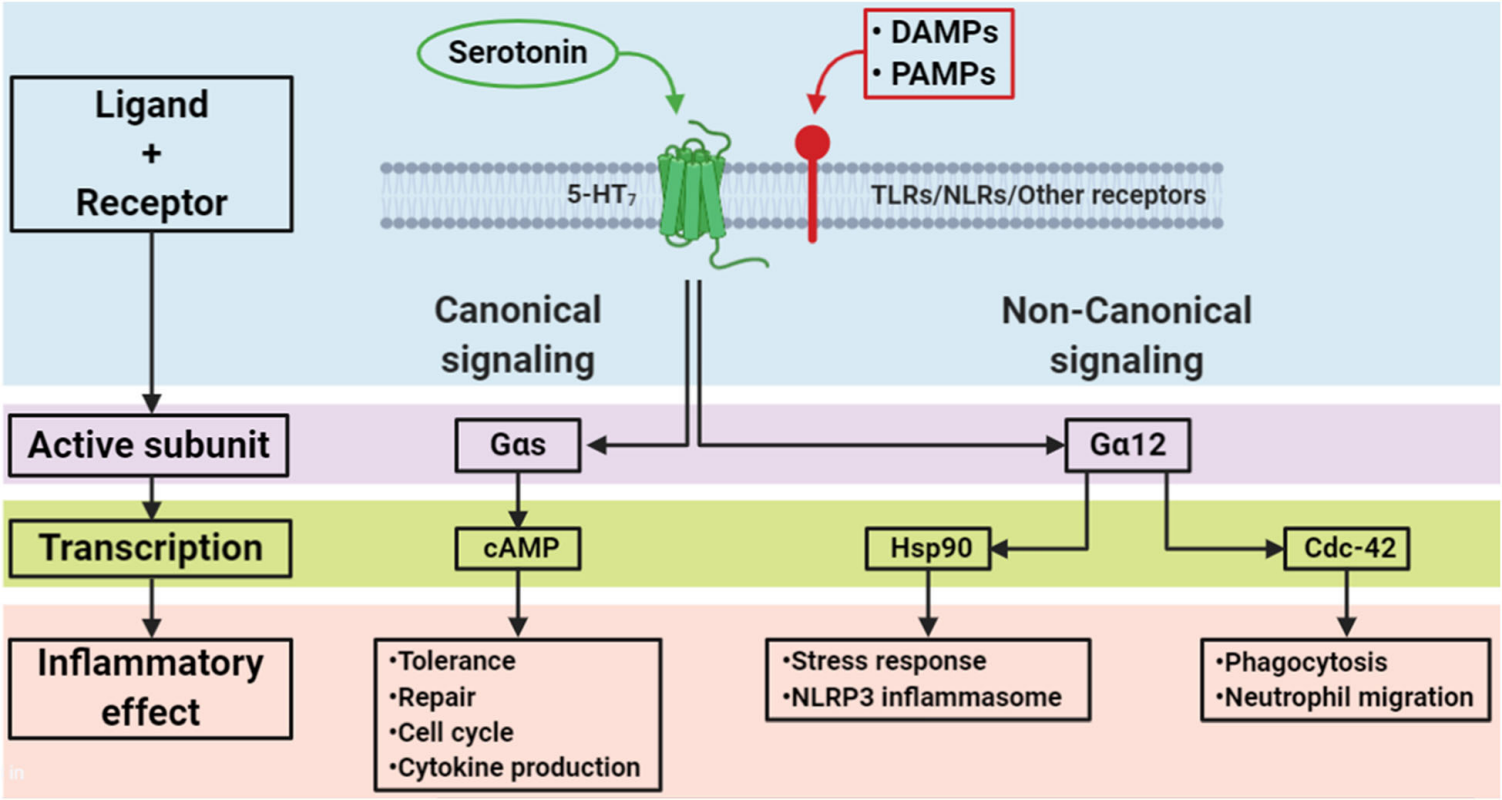
Signaling pathways downstream of 5-HT_7_ receptors

There are at least two separate signaling pathways downstream of 5-HT_7_ receptors (Fig. 1). The activation of the *canonical* signaling pathway leads to the phosphorylation of different adenylyl cyclases (AC), specially AC1 and AC8 (Baker et al. 1998). The increased activity of AC results in an increased production of cyclic adenosine monophosphate (cAMP), activation of protein kinase type A (PKA) and subsequently the phosphorylation of different target proteins like extracellular signal-regulated kinase (ERK) and Protein kinase B (also known as Akt) (Errico et al. 2001; Johnson-Farley et al. 2005). Signaling through the *non-canonical* pathway leads to activation of Gα12, whose downstream activity is mainly exerted by the Rho family of small guanosine triphosphate (GTP)-ases (Rho, Rac, cell division control protein 42 [Cdc-42]) (Guseva et al. 2014b).

Interestingly, 5-HT_7_ receptors may interact with other members of the 5-HT family of receptors. For instance, there is a well-characterized interaction between 5HT_7_ and 5-HT_1A_ receptors. 5-HT_7_ receptors can form heterodimers with 5-HT_1A_ receptors, resulting in a reduction in the activity of 5-HT_7_ receptor (Renner et al. 2005). Moreover, 5-HT_1A_ also inhibits the same signaling cascade as 5-HT_7_ receptor-mediated Gs (Zhou et al. 2019). Although to our knowledge no study has demonstrated a biological effect of this crosstalk in the immune system, this interaction could potentially explain the neutral effect of serotonin or SSRI administration.

In neuroblastoma cells, activation of 5-HT7 receptors induces the formation of filopodia via a Cdc-42-mediated pathway (Kvachnina 2005); in cultured hippocampal neurons, promotes the formation of dendritic spines and accelerates synaptogenesis; moreover, in cultured striatal and cortical neurons, activation of Gα12 leads to pronounced neurite growth via the activation of cyclin-dependent kinase 5 (Cdk5) and ERK (Speranza et al. 2013). All that suggests that 5-HT_7_ receptor signaling is critical for synaptogenesis and cell-cell communication which may also occur between non-neuronal cells, including immune ones as will be discussed below.

## Effects of 5-HT_7_ receptor signaling in immune cells

### Dendritic cells

5-HT_7_ receptors are highly expressed in mature, but not immature DCs (Idzko et al. 2004; Holst et al. 2015). 5-HT_7_ receptor activation induces DCs to release interleukin (IL)-1β and IL-8, while reducing the secretion of IL-12 and tumor necrosis factor (TNF)-α (Idzko et al. 2004). As observed in neurons, 5-HT_7_ receptor signaling in mature DCs also induces process branching and elongation via Cdc-42. While DCs do not show chemotactic response to 5-HT_7_ receptors, the non-selective high-affinity agonist 5-carboxamidotryptamine enhances velocity and distance of the chemotactic response to chemokine ligand (CCL)19 (Holst et al. 2015). The above mentioned data is summarized in Table 1.

**Table 1:** Effect of 5-HT_7_ receptor signaling on different immune cells and inflammatory conditions

Cell Type		5-HT_7_ Effect
Dendritic cells		●Induces secretion of IL-1β and IL-8; reduces secretion of IL-12 and TNF-α ●Induces process branching and elongation
Monocytes, Macrophagues, Microglia		●Pro- and anti-inflammatory ○Anti-apoptotic ○Increase in TNF-α, IL-6, Bcl-6, NF-kB ○AS-19 (agonist) decreases IL-12, TNF-α, and type 1 interferons; enhances production of TGF-β1 ○SB-269970 (antagonist) increases TNF-α and IL-12
Lymphocyte		●Concanavalin A, reserpine, and physical restrain increased expression of 5-HT_7_ ●Increase in proliferation rate, expression of CD25
Disease Model		5-HT_7_ Effect
Inflammatory Bowel Disease		●5-HT_7_ expression increased in DSS-induced colitis ●5-HT_7_ blockade/ablation results in increased severity of acute and chronic colitis ●5-HT_7_ agonists have anti-inflammatory effect
Lung Injury		●5-HT_7_ antagonists decrease lung fluid content, TNF-α, IL-6, oxidative stress in bleomycin-induced lung injury ●5-HT_7_ antagonists reduce collagen deposition, expression of TGF-β1 and procollagen type Ӏ
Central nervous system inflammation		●LP-211 (agonist) reduces neurotoxic effect of β-amyloid in a model of Alzheimer disease ●AS-19 (agonist) reduces pro-apoptotic effect of streptozotocin
Sepsis		●In LPS-induced sepsis, 5-HT7 mRNA increases in parallel to TNF-α, IL-1β, NF-κB ●LP-44 (agonist) attenuates cell injury and reduces iNOS and TNF-α ●In a CLP-induced sepsis, AS19 increases survival; reduces tissue injury, inflammatory cytokines, lung NF-κB
Liver Injury		●5-HT_7_ signaling induced during chronic liver injury ○Reduced ALT and AST levels ○Increased superoxide dismutase ○Reduced TNF-α, IL-6, TGF-β1
Soft tissue inflamation		●In carrageenan-induced paw inflammation, 5-HT7 agonists reduce cyclooxygenase mRNA expression; decrease oxidative stress, serum cytokine levels

### Monocytes and macrophages

Experimental evidence has shown an in vitro effect of 5-HT_7_ receptors on these cells, but the net effect is still incompletely understood. Monocytes treated with serotonin, or methiothepin maleate (a non-specific 5-HT_1/6/7_ receptor agonist), exhibit an inflammatory and anti-apoptotic polarization, including upregulation of TNF-α and IL-6, as well as upregulation of the transcription factors B-cell lymphoma 2 (Bcl-2), nuclear factor kappa-light-chain-enhancer of activated B cells (NF-kB); and inhibition of caspase-3. Moreover, treating monocytes with serotonin resulted in increased expression of the costimulatory molecules cluster of differentiation (CD) 40, CD80, and CD86, but not of MHC class II molecules (Soga et al. 2007). In contrast, the selective 5-HT_7_ receptor antagonist SB 269970 reverts the anti-inflammatory effect of serotonin on dextran sodium sulfate (DSS)-stimulated M2 macrophages, increasing the production of TNF-α and IL-12, while also interfering with polarization (de las Casas-Engel et al. 2013).

In human and murine macrophages, it has been recently shown that serotonin (as well as AS19, a selective 5-HT_7_ receptor agonist) decrease inflammatory priming, in part by reducing the production of IL-12, TNF-α, and type 1 interferons, as well as enhancing the production of transforming growth factor β 1 (TGF-β1). Moreover, 5-HT_7_ receptor signaling promotes pro-fibrotic gene signature in a 5-HT_7_ and PKA-dependent manner (Domínguez-Soto et al. 2017).

A role for 5-HT_7_ receptors has been experimentally observed in murine models of skin fibrosis and DSS-induced colitis. In the former, macrophage infiltration and collagen deposition are blunted by genetically or chemically interfering with 5-HT_7_ receptor signaling (Domínguez-Soto et al. 2017). Oral administration of DSS results in a well-characterized model of gastrointestinal inflammation. Interestingly, DSS also results in increased expression of 5-HT_7_ receptors in a subset of anti-inflammatory myeloid (CD11b^+^CD68^+^) cells, suggesting that myeloid expression of 5-HT_7_ receptors may also –under specific insults- attenuate the inflammatory response (Guseva et al. 2014a).

### Microglia

These, CNS-specific phagocytic mononuclear cells (Wolf et al. 2017), are produced in the yolk sac and migrate during early CNS development, before the blood brain barrier is formed. In adult life, microglia are involved in a number of homeostatic functions, including neurogenesis, synaptogenesis and synapsis remodeling, as well as neuronal apoptosis and removal (Li and Barres 2018). Microglia also actively survey the CNS for preserved molecular patterns suggestive of infection (pathogen-associated molecular patterns [PAMPs]) and tissue injury (damage-associated molecular patterns [DAMPs]) (Sankowski et al. 2015; Salter and Stevens 2017). Adult microglia express several serotonin receptors, including 5-HT_2a_, 5-HT_2b_, 5-HT_5a_, and 5-HT_7_ receptors (Krabbe et al. 2012). Microglia express at least two splice variants of the 5-HT_7_ receptor: 5-HT_7(a/b)_. In these cells, the administration of serotonin, as well as 5-carboxamidotryptamine (5-CT) induces an inflammatory priming and IL-6 production, indicating that these receptors may play a role in CNS inflammation and repair (Mahé et al. 2005).

### T cells

Lymphocytes express functional serotonin receptors (Cedeño et al. 2005; Müller et al. 2009). However, information on the role of 5-HT_7_ receptors in lymphoid cells is scant. However, preliminary evidence indicates that lymphocytes obtained from rats exposed to either concanavalin A, reserpine, or physical restraint have an increase in number of 5-HT_7_ receptor-positive lymphocytes, as well as increased expression of 5-HT_7_ mRNA (Urbina et al. 2014). Naïve splenic T cells express 5-HT_7_ receptors; their ex vivo exposure to serotonin leads to a rapid 5-HT_7_ receptor-dependent phosphorylation of ERK 1/2, increased proliferation rate, and increased expression of CD25; that response is abrogated by the 5-HT_7_ receptor antagonist SB 269970 (León-Ponte et al. 2007). Together, this suggests that 5-HT_7_ receptors play a role in T cell responses to inflammatory stimuli.

### Neutrophils

While neutrophil migration can be regulated through the effect of serotonin in other receptors, current evidence suggest that 5-HT_7_ receptor has no role on neutrophil recruitment (Rapalli et al. 2016).

### Hepatocyte response to injury

The above mentioned data is summarized in Table 1. Serotonin has been observed to play a role in liver remodeling in response to inflammatory injury, but the mechanism is incompletely understood. Current evidence suggests that hepatocyte proliferation may be regulated by 5-HT receptors at a number of levels. An in vitro study on rat hepatocytes showed that 5-HT_7_ receptor activation by serotonin dose-dependently increases cAMP and PKA signaling, whereas the pharmacological blockade by SB 269970 (a highly specific antagonist) reverted this effect, an observation with potential implications for extra-hepatic tumor seeding to the liver (Svejda et al. 2013).

## Organ and disease specific effects of 5-HT_7_

### Sepsis

In lipopolysaccharide (LPS)-induced sepsis, lung expression of 5-HT_7_ receptors increases in parallel to the increased expression of TNF-α, IL-1β, and NF-κB. Moreover, in that model, activating 5-HT_7_ receptors with LP44 attenuates LPS-induced cell injury, reducing the levels of inducible nitric oxide synthase (iNOS) and TNF-α in a dose-dependent manner (Ayaz et al. 2017).

Cecal ligation and puncture (CLP) is a well validated model of severe poli-microbial sepsis that results in acute and chronic inflammation (Valdés-Ferrer 2014; Buras et al. 2005; Valdés-Ferrer et al. 2013). Administration of a selective 5-HT_7_ receptor agonist (AS19) in a rat model of CLP-induced sepsis results in increased survival, decreased tissue injury, a reduction in circulating inflammatory cytokines (IL-1β, IL-6 and TNF-α), an increase in antioxidant mediators (superoxide dismutase and glutathione), and a reduction in lung NF-κB (Cadirci et al. 2013). The above mentioned data is summarized in Table 1.

### Inflammatory bowel disease

5-HT_7_ receptors are expressed in enteric neurons and CD11c^+^ DCs in the colon; as mentioned above, 5-HT_7_ receptor expression is significantly increased after the induction of colitis by DSS (Domínguez-Soto et al. 2017). In that model, the blockade or genetic ablation of 5-HT_7_ receptors results in increased severity of acute and chronic colitis; in contrast, 5-HT_7_ receptor agonists result in an anti-inflammatory effect (Guseva et al. 2014a; Kim et al. 2013). However, pharmacological blockade of 5-HT_7_ receptors has no effect in 2, 4, 6 trinitrobence sulfonic acid-induced colitis (Rapalli et al. 2016), suggesting that the inflammatory effect of 5-HT_7_ receptors is model-specific. More studies of the downstream-signaling mediators are needed to further understand the therapeutic potential of 5-HT_7_ receptors in experimental inflammatory bowel disease.

### Lung inflammation

Bleomycin induces experimental pulmonary fibrosis (Adamson and Bowden 1974). During the acute inflammatory phase, 5-HT_7_ receptor antagonists decrease lung fluid content, inflammatory cytokines (TNF-α, IL-6) and oxidative stress burden. In the chronic fibrogenic phase, 5-HT_7_ receptor antagonism reduces collagen deposition, and mRNA expression of TGF-β1 and procollagen type Ӏ (Tawfik and Makary 2017). In contrast, in CLP-induced sepsis, 5-HT_7_ receptor agonists reduce pro-inflammatory mediators and increase survival (Cadirci et al. 2013). Altogether, the current evidence is ambivalent regarding the usefulness of pharmacologically interfering with 5-HT_7_ receptor signaling in experimental lung injury.

### Liver disease

To our knowledge only one study has focused on the effect of 5-HT_7_ receptor signaling during chronic liver injury induced by carbon tetrachloride. There, 5-HT_7_ receptor agonists reduced alanine transaminase (ALT) and aspartate transaminase (AST) levels; increased the level of superoxide dismutase; and decreased the levels of TNF-α, IL-6 and TGF-β1. By the same token, 5-HT_7_ receptor antagonism increases cytokines levels. In their histopathological analysis, the carbon tetrachloride group showed severe vacuolar degeneration, necrosis, irregular walls of the vena centralis and lytic areas. In contrast, the administration of the 5-HT_7_ receptor agonist LP-44 partially rescued animals from liver damage. This suggests that 5-HT_7_ receptors may be a potential therapeutic target for chronic liver inflammation (Polat et al. 2017).

### Alzheimer disease (AD)

Immune activation in response to inflammatory insults is a key mediator of neurodegeneration in AD, depression, as well as other CNS disorders (Baganz and Blakely 2013; Strasser et al. 2016). Microglia engulfs and degrades β-amyloid, leading to an excessive release of inflammatory cytokines that further propagate inflammatory damage (Holmes and Butchart 2011). Also, β-amyloid binds to receptors for advances glycation end products (RAGE), resulting in further microglia activation (Querfurth and Laferla 2018). In an AD animal model, the intracerebroventricular (ICV) administration of LP-211 (a 5-HT_7_ receptor specific agonist) inhibited the neurotoxic effect of β-amyloid in hippocampus (Quintero-Villegas et al. 2018). In a rat model of stroptozotocin-induced AD, ICV administration of the 5-HT_7_ receptor-selective agonist AS19 rescued neuronal apoptosis and synaptic dysfunction (Hashemi-Firouzi et al. 2017). Altogether, available preliminary evidence derived from animal models indicates that pharmacological manipulation of the 5-HT_7_ receptor may have a niche in the treatment of AD.

### Soft-tissue inflammation

The above mentioned data is summarized in Table 1. In a carrageenan-induced paw inflammation model, 5-HT_7_ receptor agonists reduced cyclooxygenase mRNA expression, decreased oxidative stress and serum cytokine levels (Albayrak et al. 2013).

## Conclusions

5-HT_7_ receptors are widely expressed in a vast repertoire of immune and non-immune cells. The receptor has diverse -and even discrepant- roles in the immune response, probably a reflection of at least two clearly defined signaling pathways: in dendritic cells it induces the secretion of IL-1 and IL-6; in monocytes, for instance, it may either be pro- or anti-inflammatory; while in lymphocytes it increases the proliferation rate, suggesting a proinflammatory pattern.

Regarding an organ-specific effect, in CNS, soft tissue, and liver inflammatory injury, the net effect of 5-HT_7_ receptors is anti-inflammatory (reducing cell death, inflammatory cytokine release, and oxidative stress). In models of severe sepsis, 5-HT_7_ receptor agonists have resulted in a net anti-inflammatory effect. In contrast, bleomycin-induced lung injury is the only experimental model where 5-HT_7_ receptors seems to be pro-inflammatory. In experimental murine inflammatory bowel disease, the effect of 5-HT_7_ receptors has shown contradictory results.

While our understanding of the connection between 5-HT_7_ receptors and inflammation is still limited, significant advances have emerged in the past two decades. Nonetheless, current evidence suggests that pharmacological interventions targeting 5-HT_7_ receptors may be potentially useful for treating inflammatory conditions.

## Data Availability

Not applicable (other than the referenced publications).

## Abbreviations

5-HT: Serotonin
5-HTRs: 5-HT receptor family
AC: Adenylyl cyclase
ALT: Alanine transaminase
AST: Aspartate transaminase
Bcl-2: B-cell lymphoma 2 transcription factor
CA: *Cornu ammonis* region of the hippocampus
cAMP: Cyclic adenosine monophosphate
CCL: Chemokine ligand
CD: Cluster of differentiation
Cdc-42: Cell division control protein 42
Cdk5: Cyclin-dependent kinase 5
CLP: Cecal ligation and puncture
CNS: Central nervous system
DAMPs: Damage-associated molecular patterns
DCs: Dendritic cells
DSS: Dextran sodium sulfate
ERK: Extracellular signal-regulated kinase
GI: Gastrointestinal
GTP: Guanosine triphosphate
ICV: Intracerebroventricular
IL: Interleukin
iNOS: Inducible nitric oxide synthase
LPS: Lipopolysaccharide
mRNA: Messenger RNA
NF-kB: Nuclear factor kappa-light-chain-enhancer of activated B cells
PAMPs: Pathogen-associated molecular patterns
PKA: Protein kinase type A
TGF-β1: Transforming growth factor beta 1
TNF: Tumor necrosis factor
